# Les myélopathies cervicarthrosiques: résultat clinique et radiologique de la chirurgie sur une série de 135 patients opérés au service de neurochirurgie CHU Avicenne

**DOI:** 10.11604/pamj.2014.19.29.4481

**Published:** 2014-09-12

**Authors:** Ben Ousmanou Djoubairou, Moussé Nabil, Claire Karekezi, Seylan Diawara, Nizar El Fatemi, Rachid Gana, Najia El Abbadi, Moulay Rachid Maaqili

**Affiliations:** 1 Service de Neurochirurgie, Faculté De Médecine et Pharmacie de Rabat, Université Mohamed V Souissi, CHU Avicenne, Rabat, Maroc

**Keywords:** Myélopathie, cervicarthrose, chirurgie, lordose cervicale, évolution, Myelopathy, Cervical spondylosis, surgery, cervical lordosis, evolution

## Abstract

La myélopathie cervicarthrosique est un syndrome clinique en relation avec la diminution des dimensions du canal rachidien, la cervicarthrose est l’étiologie principale après 50 ans. L'objectif du traitement est de rétablir les dimensions du canal rachidien cervical. Le choix de la technique chirurgicale sera guidé par l'analyse des signes cliniques, imageries, pré opératoire en fonction de laquelle sera pratiquée soit la voie antérieure, postérieure, ou exceptionnellement la voie combinée. Notre étude a pour but dans un premier temps d’évaluer à long terme les résultats cliniques et radiologiques de la chirurgie ensuite répondre à cette préoccupation: La lordose cervicale pré opératoire et postopératoire sont t-elles des facteurs de bon pronostic? Nous rapportons une étude rétrospective entre 2000 et 2013 portant sur 135 patients opérés dans notre formation et remplissant les critères inclusions. La collecte des données s'est faite en s'aidant du dossier médical des patients (échelle d'Association des orthopédistes Japonais), Imagerie (Radio, TDM, IRM), mesure de l'angle de courbure rachidienne en pré et postopératoire, ceci dans le but d’évaluer à long terme les résultats clinique et radiologique de la chirurgie. Ont été inclus dans notre étude 135 patients, 82 Hommes (60%), 53 femmes (40%) avec un âge moyen de 52 ans, ayant consulté pour des motifs divers (Névralgies cervicobrachiales, lourdeur des membres, troubles génito-sphinctériens). Soixante cinq patients (48%) ont bénéficié d'un abord antérieur (dissectomie, cloward, somatotomie médiane), 64 patients (47%) ont été opérés par voie postérieure (laminectomie de 1 à 3 niveaux) et 6 patients (5%) ont bénéficié d'un abord combiné dans un délai moyen de 3 mois devant la persistance des symptômes. Le niveau cervical le plus touché était C5C6 suivie de C4C5. L’évolution globale de nos patients était favorable dans 58% des cas, stationnaire dans 41% des cas et 1% d'aggravation. Soixante patients ayant présentés une amélioration en postopératoire avaient une courbure rachidienne en lordose, contre 17 patients en raideur et aucun patient en cyphose (p < 0.05). En définitive, la myélopathie est une pathologie fréquente dans la pratique neurochirurgicale, le diagnostic s'est beaucoup amélioré grâce à l'avènement de IRM, plusieurs voies d'abords sont utilisées en fonction des données cliniques et d'imageries, l’évolution reste favorable si la prise en charge est précoce avant l'apparition des déformations importantes de l'alignement sagittal du rachis.

## Introduction

La myélopathie cervicarthrosique est un syndrome clinique en relation avec la diminution des dimensions du canal rachidien, la cervicarthrose est l’étiologie principale après 50 ans [1]. Le traitement a pour objectif de rétablir les dimensions du canal rachidien cervical. Le choix de la technique chirurgicale sera guidé par l'analyse des signes cliniques, imageries, pré opératoire en fonction de laquelle sera pratiquée soit la voie antérieure, postérieure, ou exceptionnellement la voie combinée.

## Méthodes

Nous rapportons une étude rétrospective entre 2000 et 2013 portant sur 135 patients opérés dans notre formation et respectant les critères inclusions suivants: Patient opéré pendant la période définie de notre étude et ayant bénéficié d'un suivie d'au moins 6 mois en post- opératoire; Patient ayant un dossier complet permettant de ressortir les éléments de l’échelle de l'association des orthopédistes japonais (motricité, sensibilité, troubles sphinctériens). La collecte des données s'est faite en s'aidant du dossier médical des patients, il a fallu remplir un questionnaire en pré et post- opératoire, contenant les éléments de l’échelle de l'Association des orthopédistes Japonais (AOJ), les donnés de l'imagerie (Radio, IRM), la mesure de l'angle de courbure rachidienne en pré et post- opératoire, ceci dans le but d’évaluer à long terme les résultats cliniques et radiologiques de la chirurgie.

**Clinique:** Nos patients ont bénéficié systématiquement d'un bilan comprenant des radiographies standard face et profil, une IRM cervicale et pour certains d'une TDM cervicale, un EMG en cas de névralgie cervicobrachial et enfin le bilan pré opératoire suivie d'une consultation pré anesthésique. Les patients opérés ont été vu systématiquement à 3 mois et 6 mois après l'acte chirurgical puis de façons irrégulières selon la symptomatologie résiduelle. Nous avons remplie pour chaque patient un questionnaire structuré selon l’échelle de AOJ ressortissant l′état fonctionnel en pré et post opératoire pour jugé de l’évolution clinique des symptômes.

**Analyse radiologique:**Les mesures pré et post opératoire ont été réalisée sur des radiographies standard du rachis cervical profil en position neutre par la méthode de Cobb: le rachis est en lordose lorsque l'angle est supérieur ou égale a +5; en rectitude quand l'angle est compris entre +5 et -5; en cyphose lorsque l'angle est inférieure ou égale à -5. Par ailleurs nous avons vérifié la concordance avec la méthode globale c'est-à-dire une ligne joignant le point le plus postérieur du plateau inférieur de C2 à un point similaire sur C7: Si un corps vertébral coupe la ligne, le rachis est en cyphose; si la partie postérieure des corps vertébraux affleure la ligne, le rachis est en rectitude; Sur un rachis en lordose, les corps vertébraux sont en avant de la ligne.

**Chirurgie:**Les dossiers médicaux des patients étaient discutés en staff, la voie d'abord retenue de façon collégiale en s'aidant des données cliniques et de l'imagerie: Voie antérieure et ses variantes (Cloward, Somatotomie médiane, Dissectomie) avec greffon iliaque (autogreffe), allogreffe (avec ou sans substitut osseux); Voie postérieure (laminectomie); Voie combinée (sténoses majeures avec lésions antérieures et postérieures, mais surtout pour les résultats incomplet d'une décompression antérieure ou postérieure ou de récidive des signes cliniques après une phase de récupération).

**Statistique:**les résultats sont exprimés sous forme de moyenne et écart-types. Une valeur de *p*≤0,05 est considérée comme significative. Les données ont été analysées à partir du logiciel SPSS.

## Résultats

**Epidémiologie:** La taille de notre échantillon était de 135 patients, 82 hommes et 52 femmes d'un âge compris entre 29-74 ans avec une moyenne d’âge de 52 ans.

**Clinique:** Dominé par une atteinte motrice allant de la simple tétraparésie à une tétraplégie (65 cas), NCB (55 cas), cervicalgie et troubles génitosphintériens (15 cas). Le délai entre début de la symptomatologie et l'intervention chirurgicale allait de 6 à 30 mois avec une moyenne de 15 mois.

**Chirurgie:** Voie antérieure: Soixante-cinq patients ont bénéficié d'un abord antérieur parmi lequel 40 ont bénéficié de la mise en place d'un greffon iliaque et 25 d'un allogreffe. Les disques concernés étaient: C4C5 (39 cas), C5C6 (43 cas), C6C7 (18 cas). Le nombre de dissectomie variait de 1 à 4, deux patients ont été opérés de 4 niveaux, quarante patients ont été opérés de 3 niveaux, 20 patients de 2 niveaux, et seulement 3 patients d'un niveau. Pas d'infection de la plaie opératoire, ni de décès dans notre série, 3 patients ont été repris pour migration du greffon objectivé surtout chez les patients ayant une autogreffe. - Voie postérieure: Soixante- quatre patients ont été opéré par voie postérieure pour laminectomie d'un ou de 2 niveaux, les niveaux concernés C3, C4, C5, C6. La laminectomie la plus réalisée était celle de C5 (34 cas). La majorité des patients étaient opérés pour deux niveaux (60 cas) et seulement 4 malades ont bénéficié d'une laminectomie d'un niveau (canal cervical constitutionnellement étroit). Pas d'infection de la plaie opératoire, cependant ont a eu 3 cas de brèches de la dure-mère qui ont été réparé. Voie combinée Réalisé de façon exceptionnel chez 6 patients chez qui ont a noté une aggravation secondaire après une récupération brève en postopératoire ou apparition d'une symptomatologique purement sensitive ayant nécessité une foraminotomie par voie postérieure.

### Evolution

**Evolution Clinique:** Voie antérieure: 43 patients ce sont améliorés, 20 stationnaires, 2 se sont aggravés; Voie postérieure: 34 amélioré, 30 stationnaires restant dépendant du traitement médical; Voie combiné: 1 patient amélioré, 5 stationnaires par rapport à la symptomatologie initiale.

**Evolution Radiologique:** Voie antérieure: En préopératoire, 41 patients présentaient une courbure cervicale en lordose (63%), parmi lesquels 26 patients sont restés en lordose alors que 15 patients ont évolué vers une raideur du rachis cervical. Sur vingt patients en raideur cervicale préopératoire, 12 sont passés en lordose et 8 sont restés en raideur cervicale. Enfin sur 4 patients en cyphose 2 sont passés en raideur cervicale tandis que 2 sont restés en raideur (Tableau 1). Voie postérieure: En préopératoire, 32 patients étaient en lordose cervicale, parmi lesquels 20 sont restés en lordose cervicale en postopératoire, 11 patients sont passés en raideur cervicale et seulement 1 patient en cyphose. Sur 30 patients en raideur cervicale préopératoire, 13 sont restés en raideur cervicale, 15 sont passés en lordose cervicale et 2 cas de cyphoses cervicales ont été retrouvés. Enfin les 2 patients en cyphose cervicale sont restés en cyphose avec tendance à aggravation de l'angulation (Tableau 1). Voie combinée: nous n'avons pas noté de changement de courbure chez les 6 patients qui ont bénéficiés d'un abord combiné.

**Table 1:** Alignement sagittal du rachis cervical en pré et postopératoire (voie antérieure et postérieure)

Alignement préopératoire			Alignement		Postopératoire		(%)	
	Cyphose		Raideur		Lordose		Total	
Voie Abord	Ant	Post	Ant	Post	Ant	Post	Ant	Post
Cyphose	2	2	2	-	-	-	4 (6%)	2 (3%)
Raideur	-	2	8	13	12	15	20 (31%)	30 (47%)
Lordose	-	1	15	11	26	20	41 (63%)	32 (50%)
Total	2 (3%)	5 (8%)	25 (38%)	24 (37%)	38 (59%)	35 (55%)	65	64

**Ant**= Antérieure, **Post**= Postérieure

**Figure 1:**
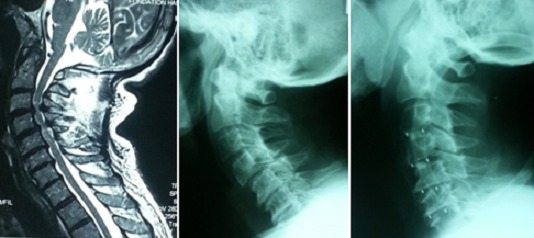
IRM préopératoire coupe sagittale en séquence pondérée T2 objectivant une myélopathie cervicarthrosique avec hyper signal médullaire en regard de C5, radiographie préopératoire et à 12 mois postopératoire d'un patient de 52 ans ayant bénéficié d'une dissectomie de 4 niveaux avec mise en place de 4 cages

**Figure 2:**
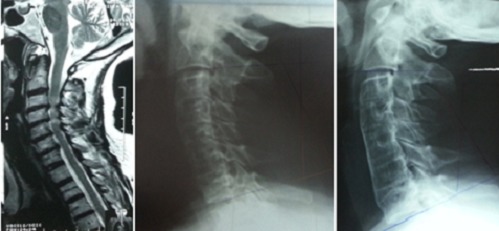
IRM préopératoire coupe sagittale en séquence pondérée T2 objectivant une myélopathie cervicarthrosique, radiographie préopératoire et à 6 mois postopératoire d'un patient de 58 ans ayant bénéficié d'une dissectomie de 3 niveaux avec mise en place de 3 greffons iliaques

**Evolution clinique et profil radiologique postopératoire:** Voie antérieure: Quarante- trois patients ce sont améliorés en postopératoire parmi lesquels 30 patients en lordose cervicale postopératoire, 13 seulement en raideur et aucun patient en cyphose (Figure 1, Figure 2). Vingt patients sont restés stationnaire parmi lesquels 12 cas de raideur du rachis cervical, 8 cas de lordose rachidienne. Deux patients ce sont aggravés et ont gardé une courbure du rachis cervicale en cyphose (Tableau 2). Voie postérieure: Trente-quatre patients ce sont améliorés parmi lesquels 30 ont une courbure rachidienne en lordose et 4 en raideur. 30 patients sont restés stationnaires sur le plan clinique (20 cas de raideur et 5 cas de cyphose cervicale), cependant les patients ayant une évolution clinique stationnaire avaient en plus un angle de mobilité du rachis bas (en moyenne 29°) contrairement aux groupes qui s'est amélioré (42°). Soixante patients ayant présentés une amélioration en postopératoire avaient une courbure rachidienne en lordose, contre 17 patients en raideur et aucun en cyphose (p < 0.05)

**Table 2:** Alignement sagittal et évolution clinique

Alignement postopératoire			Amélioration		Clinique		(AOJ)	
	Amélioré		Stationnaire		Aggravé		Total	
Voie Abord	Ant	Post	Ant	Post	Ant	post	Ant	Post
Cyphose	-	-	-	5	2	-	2 (3%)	5 (8%)
Raideur	13	4	12	20	-	-	25(38%)	24 (37%)
Lordose	30	30	8	5	-	-	38 (59%)	35(55%)
Total	43 (66%)	34 (53%)	20 (31%)	30 (47%)	2 (3%)	-	65	64

**Ant**= Antérieure, **Post**= Postérieure, **AOJ**= Association des orthopédistes Japonais

## Discussion

La myélopathie par cervicarthrose est une pathologie fréquente à partir de 50 ans mais peut survenir plus précocement à partir de 30 ans, l'homme est plus touché que la femme [1], l’âge moyen des patients de notre série était de 52 ans avec une nette prédominance masculine. Le traitement chirurgical avait pour but de rétablir les dimensions du canal rachidien cervical, le choix de la technique est fonction du siège et de l’étendue des lésions: La laminectomie et ses variantes pour les sténoses constitutionnelles étendues, abords antérieures pour les compressions discarthrosiques, La somatotomie médiane longitudinale proposée pour des lésions intéressant plus de deux étages associée ou non à une greffe (autogreffe ou allogreffe) avec une ostéosynthèse pour éviter la cyphose postopératoire [2, 3]. Brunon en 1997 rapporte que la voie antérieure est réalisé dans près de 80% des cas par les neurochirurgiens européens francophones, la voie postérieure réalisée dans 15% des cas et la voie combinée réalisée de façon exceptionnelle [4].

Dans notre série la voie postérieure et antérieure représentait respectivement 48% et 47%, cette constatation est due au fait que de nombreux patients opérés par voie antérieure ne respectaient pas les critères d'inclusions (patients perdus de vue après 3 mois de suivie postopératoire, l'absence des radiographies de contrôles postopératoire, dossiers incomplets). Cependant Arnold H. et all. En 1993 rapportait une série de 70 patients dont 44 cas de laminectomie, 19 cas d'abord antérieur, et 7 cas d'abord combiné [5]. L'atteinte du rachis cervicale par la myélopathie cervicarthrosique intéresse la partie mobile du rachis cervicale, dans notre série la majorité des lésions étaient localisées au niveau du disque C5C6 (43 cas) pour ce qui est des patients abordés par voie antérieur alors que la laminectomie par voie postérieure intéressait la lame de C5 (34 cas). L’évolution clinique globale dans notre série était favorable dans 58%, selon la voie d'abord utilisée 66% des patients opérés par voie antérieure ce sont améliorés (échelle de AOJ) contre 53% par voie postérieure. Arnold et all rapporte sur une période de 6 mois une évolution favorable dans 77% pour les patients ayant bénéficiés d'une laminectomie contre 90% pour les patients opéré par voie antérieure, cependant on note une détérioration secondaire sur le plan clinique à la huitième année surtout dans le groupe ayant bénéficié une laminectomie par voie postérieure. Les auteurs pensent que ceci s'explique par l'installation d'une cyphose progressive en rapport soit avec l’évolution naturelle de la maladie ou la déstabilisation du rachis par l'acte chirurgical. L'apparition d'une cyphose post opératoire a été rapportée par plusieurs auteurs, elle est de 8,8% pour les abords antérieure [6] et varie entre 1 à 30% pour les abords postérieur [7, 8, 2]. Dans notre série ont retrouve 3% de cyphose pour le groupe opéré par voie antérieure contre 8% par voie postérieure. L'apparition d'une cyphose paraît plus en relation avec une insuffisance de la musculature nucale, raison pour laquelle il est important de réalisé une rééducation postopératoire précoce. Le port à long terme d'un collier cervical pourrait favoriser l'atrophie des muscles paravertébraux et augmenterait le risque de cyphose [9].

Les patients ayant une courbure rachidienne en lordose en préopératoire et en postopératoire ont une meilleure amélioration clinique à court et à moyen terme dans notre série soit 66% par voie antérieure contre 53% par voie postérieure [10–12], d'où l'hypothèse émise par Batzdorf selon laquelle la restauration de la lordose du rachis cervicale et l'espace libéré par la laminectomie entrainerait une migration de la moelle cervicale vers l'arrière à distance des ostéophytes. Cette théorie a été réfuté par Mikawa et al en 1987, sur une série de 64 patients opéré par voie postérieure avec un suivie radiologique de 2 ans qui objectivait 36% de modification de la statique du rachis cervicale et l'apparition de 14% de cyphose. Cependant cette constatation n'avait pas été corrélé à un mauvais pronostic ni à un déficit neurologique [13, 14]. Kaptain aborde dans le même sens et conclut que toute entreprise destinées à empêcher la déformation du rachis cervicale pourrait être ainsi inutile [8]. L'alignement sagittal du rachis cervical proche de l'anatomie normale aussi bien en préopératoire qu'en postopératoire était un facteur de bon pronostic dans notre série. La cyphose postopératoire apparait chez 3% des patients opérés par voie antérieure contre 8% par voie postérieure. Ce taux est considérablement revu à la baisse dans les séries où les patients ont bénéficié d'une rééducation précoce des muscles nucaux [9].

## Conclusion

La myélopathie est une pathologie fréquente dans la pratique neurochirurgicale, le diagnostic s'est beaucoup amélioré grâce à l'avènement de IRM, plusieurs voies d'abord sont utilisées en fonction des données cliniques et d'imageries, l’évolution reste favorable si la prise en charge est précoce avant l'apparition des déformations importantes de l'alignement sagittal du rachis.
